# Variation and significance of serum microRNA-21 level in pediatric pulmonary artery hypertension associated with congenital heart disease

**DOI:** 10.3389/fcvm.2024.1424679

**Published:** 2024-09-05

**Authors:** Yanming Shen, Dongshan Liao, Wenlin Shangguan, Liangwan Chen

**Affiliations:** ^1^ Cardiac Surgery, Fujian Medical University, Fuzhou, Fujian, China; ^2^ Department of Cardiovascular Surgery, Union Hospital, Fujian Medical University, Fuzhou, Fujian, China; ^3^ Key Laboratory of Cardio-Thoracic Surgery, (Fujian Medical University), Fujian Province University, Fuzhou, Fujian, China; ^4^ Thoracic and Cardiovascular Surgery, Fuzhou Changle District People’s Hospital, Fuzhou, Fujian, China

**Keywords:** congenital heart disease, pulmonary artery hypertension, *miR-21*, pediatric critical illness score, clinical significance

## Abstract

**Objective:**

This study strives to the variation and significance of microRNA-21 (*miR-21*) in children with congenital heart disease (CHD)-related pulmonary artery hypertension (PAH).

**Methods:**

Children with CHD (*n* = 179) were selected as subjects, including 101 children without PAH and 78 children with PAH. All children underwent general data collection, laboratory examination, echocardiography and cardiac catheterization. After detection of serum *miR-21* expression, the predictive value and the impacts of serum *miR-21* for PAH and postoperative critical illness were analyzed.

**Results:**

Serum creatine kinase isoenzyme (CK-MB), B-type natriuretic peptide (BNP) and *miR-21* were elevated, but ejection fraction (EF) and cardiac index (CI) were decreased in the CHD-PAH group. Serum *miR-21* assisted in predicting PAH in CHD children, with the area under curve (AUC) of 0.801 (95% CI of 0.735∼0.857), a cut-off value of 2.56, sensitivity of 73.08, and specificity of 72.28%. Serum *miR-21* in children with CHD-PAH was correlated with clinicopathological indicators such as systolic pulmonary artery pressure, mean pulmonary arterial pressure, BNP and CI. Serum *miR-21* helped predict the development of postoperative critical illness in children with CHD-PAH, with an AUC of 0.859 (95% CI: 0.762–0.927, cut-off value: 4.55, sensitivity: 69.57%, specificity: 92.73%). Increased serum *miR-21* was an independent risk factor of postoperative critical illness in children with CHD-PAH.

**Conclusion:**

Serum *miR-21* was upregulated in children with CHD-PAH, which may serve as a predictive biomarker for the onset of PAH and postoperative critical illness in CHD children.

## Introduction

Congenital heart disease (CHD) is a disease of abnormal fetal cardiac and cardiovascular development that affects approximately 1% of infants born each year (1). Patients with CHD are at a high risk of developing pulmonary artery hypertension (PAH) (2). Chronic right ventricular pressure overload makes children with PAH associated with CHD (CHD-PAH) prone to right ventricular hypertrophy, volume overload and failure, eventually evolving into right ventricular dysfunction (3). Noticeably, the development of PAH may substantially increase the mortality in patients with CHD (4). Unfortunately, children with CHD are more susceptible to developing PAH instead of adults with CHD (5).

Cardiac catheterization, a gold standard for definitive diagnosis of PAH so far, enables direct evaluation of pulmonary hemodynamics and implementation of vasoreactivity test (6). However, this approach is likely to put patients at risk of complications, such as arrhythmia, hypertensive crisis, pulmonary embolism and even death (7, 8). In contrast, echocardiography (echo) is a non-invasive method that can be extensively used in patients with CHD-PAH (3), but the accuracy of echo is affected by various factors, such as the experience of operators, equipment quality and patient compliance with examinations, to name a few. At present, there is a lack of specific, economic and non-invasive methods for CHD-PAH screening.

MicroRNAs (miRNAs), small non-coding RNA, have garnered significant research attention in recent years as they participate in the pathophysiological process of various cells through their regulation of gene expression (9). Dysregulation of miRNAs appears to implicate in various heart diseases including CHD (10–13). *miR-21* is an encoding gene in the fragile site FRA17B within the human 17q23.2 chromosomal region, and it has been identified by previous studies to be associated with cardiac hypertrophy, heart failure, and myocardial infarction (14, 15). Reportedly, increasingly expressed *miR-21* was found in myocardial fibroblasts and associated with vascular endothelial disorder and pulmonary vascular remodeling (16, 17). Accordingly, it is assumed that abnormal *miR-21* expression may be related to the development of PAH and postoperative crisis in children with CHD-PAH. More importantly, a recent report disclosed that upregulation of *miR-21* in the early stage of PAH results in right ventricular hypertrophy, whereas in the late stage of PAH, right ventricular dysfunction is caused by its downregulation, which triggers a biphasic regulation of cardiac remodeling and cardiomyocyte apoptosis (18). In a pressure overload-induced PAH model in sheep, *miR-21* was identified as a critical factor for the occurrence of right ventricular hypertrophy and dysfunction (19). All this information indicates that *miR-21* may show great importance in children with CHD-PAH, but the number of studies focusing on the change of *miR-21* and its clinical significance in children with CHD-PAH is limited. This study is designed to discover the change of serum *miR-21* and its significance in children with CHD-PAH, providing a novel insight for clinical diagnosis and treatment of CHD-PAH.

## Materials and methods

### Ethics statement

This study was reviewed and granted by the Ethics Committee of Union Hospital, Fujian Medical University and complied with the *Declaration of Helsinki*. Written informed consent was obtained from the guardian of each enrolled participant.

### Participants

A total of 256 children with CHD admitted to the hospital from February 2020 to May 2023 were enrolled, and 179 children with CHD were selected as the study subjects according to the inclusion and exclusion criteria. Based on the occurrence of PAH, the children with CHD were stratified into CHD (without CHD-related PAH, *n* = 101) and CHD-PAH groups (with PAH associated with CHD, *n* = 78). General information, laboratory findings, echo results, and cardiac catheterization results were collected from all children with CHD. Another 65 healthy participants who had normal physical examination results were chosen as the control group.

### Inclusion and exclusion criteria

Diagnostic criteria for CHD: examined by echo and confirmed by vascular CT or cardiovascular angiography.

Diagnostic criteria for CHD-related PAH: diagnosed by cardiac catheterization with mean pulmonary arterial pressure (mPAP) ≥ 20 mmHg by right heart catheterization when the child was resting (20).

Inclusion criteria: 1. age < 12 years old; 2. all children underwent cardiac catheterization before intervention; 3. treated with minimally invasive cardiac surgery for the first time; 4. complete clinical data.

Exclusion criteria: 1. combination of severe infection, tumor, severe cardiopulmonary insufficiency, diabetes mellitus, systemic hypertension, cardiomyopathy, hepatic insufficiency or renal failure and bleeding tendency; 2. Presence of hematological system diseases, connective tissue diseases, and other respiratory system diseases.

### Case information collection

The medical records of children with CHD were consulted to collect age, sex, heart rate (HR), arterial systolic arterial pressure (SBP), arterial diastolic arterial pressure (DBP), ejection fraction (EF), cardiac index (CI), left ventricular end-diastolic diameter (LVEDd), systolic pulmonary artery pressure (sPAP), diastolic pulmonary artery pressure (dPAP), mPAP, serum creatine kinase isoenzyme (CK-MB), B-type natriuretic peptide (BNP), pulmonary vascular resistance (PVR), and other information.

### RNA isolation and reverse transcription polymerase chain reaction (Rt-PCR)

All children with CHD underwent minimally invasive cardiac surgery and were subjected to elbow venous blood sampling (5 ml) using a non-anticoagulant vacuum tube. After centrifugation for 15 min (radius 8 cm, 4,000 r/min), the serum was preserved in a refrigerator at −80°C for testing. An miRNA extraction kit (Beijing Tiangen Biotechnology Co., Ltd., Beijing, China) was used to extract miRNAs which were reverse transcribed to cDNA with a reverse transcription kit (TaKaRa Bio, Tokyo, Japan). RT-PCR method and MX3000PPCR instrument from USA Agilent were adopted to amplify *miR-21* target segments. With U6 snRNA as the housekeeping gene, the RT-PCR experiment was performed with a reaction system of 20 μl under the conditions of pre-denaturation at 95°C for 10 min, 95°C for 30s and 60°C for 30 s, for a total of 40 cycles. The ratio of the target gene level to the U6 level was calculated and the relative level of *miR-21* was expressed by 2^−*ΔΔ*Ct^. See Table 1 for specific primer sequences.

**Table 1:** Primer sequences used for RT-PCR.

Gene	Forward (5′-3’)	Reverse (5′-3’)
*miR-21*	ACACTCCAGCTGGGTAGCTTATCAGACTGA	CTCAACTGGTGTCGTGGAGTCGGCAATTCAGTTGA
*U6*	CTCGCTTCGGCAGCACA	CTCGCTTCGGCAGCACA

RT-PCR, reverse transcription polymerase chain reaction.

### Postoperative critical illness score

Pediatric critical illness score (PCIS) was adopted to evaluate 10 items: HR, blood pressure, serum creatinine, urobilin, partial arterial oxygen pressure, serum potassium level, serum sodium level, hemoglobin level, respiratory system status and gastrointestinal system status (21). The maximum abnormal value of each index was recorded within 24 h after the children were admitted to the monitoring room, and the corresponding scores were summed up to get the total score out of 100 points. The lower the score, the more critical the condition. A PCIS score of > 80 points represents a non-critical case, a PCIS score of 71–80 points indicates a critical case, and a PCIS score of ≤ 70 points represents a very critical case.

### Statistic analysis

Data were statistically analyzed and graphed using SPSS 21.0 statistical software (SPSS, Inc, Chicago, IL, USA) and GraphPad Prism 6.0 software (GraphPad Software Inc., San Diego, CA, USA). The Kolmogorov-Smirnov test was used to test for normal distribution, and measurements conformed to normal distribution were expressed as mean ± standard deviation. Comparisons between two groups were performed using the independent sample *t*-test, whereas multiple groups were analyzed by one-way analysis of variance (ANOVA), and *post-hoc* tests were performed using Tukey's multiple comparisons test. Non-normally distributed measurements were expressed as quartiles, i.e., median (minimum, maximum), and comparisons between groups were made using the Mann-Whitney *U*-test; count data were expressed as case numbers and percentages, and comparisons between groups were made using the chi-square test. A multi-factor logistic regression model was established to analyze the impacts of changes in serum *miR-21* level on the occurrence of PAH and postoperative critical illness in children with CHD, and the Enter method was selected to screen the independent variables. ROC curve was used to analyze the predictive value of serum *miR-21* level on the occurrence of PAH and postoperative critical illness in children with CHD, and the cut-off value and the area under the ROC curve (AUC) of serum *miR-21* were obtained. *P* was a two-sided test, and the difference was considered statistically significant at *P* < 0.05.

## Results

### Comparison of baseline data between the three groups

This study selected 179 children with CHD admitted to the hospital from February 2020 to May 2023 as the study subjects and stratified them into CHD (without CHD-related PAH, *n* = 101) and CHD-PAH groups (with CHD-related PAH, *n* = 78). Another 65 healthy subjects were included as the control group. No significant difference was found in sex, age, body mass index (BMI), HR, SBP, DPB, LVEDd and other indexes among the three groups (all *P* > 0.05, Table 2), and the CHD group had no significant difference in disease type and mPAP compared with the CHD-PAH group (all *P* > 0.05, Table 2). However, the EF, BNP and CK-MB levels were substantially different among the three groups (all *P* < 0.05, Table 2), and the children in the CHD-PAH group had higher BNP and CK-MB levels and lower EF. Moreover, sPAP, mPAP, dPAP, and PVR were higher in the CHD-PAH group than those in the CHD group, but the CI in the CHD-PAH group was decreased compared with the CHD group (all *P* < 0.05, Table 2).

**Table 2:** Baseline comparisons of clinical characteristics.

Items	Control (*n* = 65)	CHD (*n* = 101)	CHD-PAH (*n* = 78)	*P*-*value*
Sex (male/female)	35/30	41/60	36/42	0.247
Age (year)	5 (2,10)	6 (2,11)	6 (2,10)	0.474
BMI (kg/m^2^)	13.48 ± 2.42	13.46 ± 2.15	13.44 ± 2.14	0.996
CHD type [*n*(%)]				
VSD	–	60 (59.41)	47 (60.26)	0.988
ASD	–	34 (33.66)	26 (33.33)	
PDA	–	7 (6.93)	5 (6.41)	
HR (beats/min)	95.28 ± 3.17	94.89 ± 3.30	94.86 ± 3.04	0.685
SBP (mmHg)	104.89 ± 9.53	105.81 ± 9.69	103.46 ± 12.29	0.337
DBP (mmHg)	64.94 ± 4.93	64.37 ± 6.29	62.77 ± 6.19	0.070
EF (%)	68.42 ± 3.54	69.26 ± 4.59	63.88 ± 6.93	<0.001
LVEDd (mm)	34 (30,44)	36 (29,44)	34 (28,45)	0.198
sPAP (mmHg)	–	24.70 ± 3.53	55.08 ± 9.99	<0.001
mPAP (mmHg)	–	16.19 ± 2.13	36.04 ± 5.42	<0.001
dPAP (mmHg)	–	11.94 ± 2.83	26.53 ± 6.71	<0.001
mRAP (mmHg)	–	5.00 (4.00,6.00)	5.50 (3.00,8.00)	0.604
PVR (WU)	–	1.98 ± 0.40	3.47 ± 1.15	<0.001
CI (L min^−1^m^−2^)	–	7.63 (5.56,9.09)	6.25 (4.43,8.73)	<0.001
BNP (pg ml^−1^)	54.41 (25.08,79.10)	138.19 (45.42,295.46)	405.76 (121.02,714.14)	<0.001
CK-MB (U L^−1^)	11.23 ± 3.15	21.33 ± 3.19	25.13 ± 4.38	<0.001

BMI, body mass index; CHD, congenital heart disease; VSD, ventricular septal defect; ASD, atrial septal defect; PDA, patent ductus arteriosus; HR, heart rate; SBP, systolic blood pressure; DBP, diastolic blood pressure; EF, ejection fraction; LVEDd, left ventricular end-diastolic diameter; sPAP, systolic pulmonary arterial pressure; mPAP, mean pulmonary arterial pressure; dPAP, diastolic pulmonary arterial pressure; mRAP, mean right atrial pressure; PVR, pulmonary vascular resistance; CI, cardiac index; BNP, B-type natriuretic peptide; CK-MB, creatine kinase-MB. Measurements are expressed with case numbers and percentages. Comparisons between groups were performed using chi-square test. Measurements that conformed to normal distribution were expressed as average ± standard deviation; comparisons between two groups were conducted using an independent sample *t*-test and those among multiple groups using one-way analysis of variance. Tukey's multiple comparisons test was adopted for *post-hoc* test. Non-normally distributed measurements were expressed as quartiles and Mann-Whitney *U*-test was used for comparisons between groups.

### Children with CHD-PAH had higher serum *miR-21* levels, which assisted in predicting the occurrence of PAH in children with CHD

**Figure 1:**
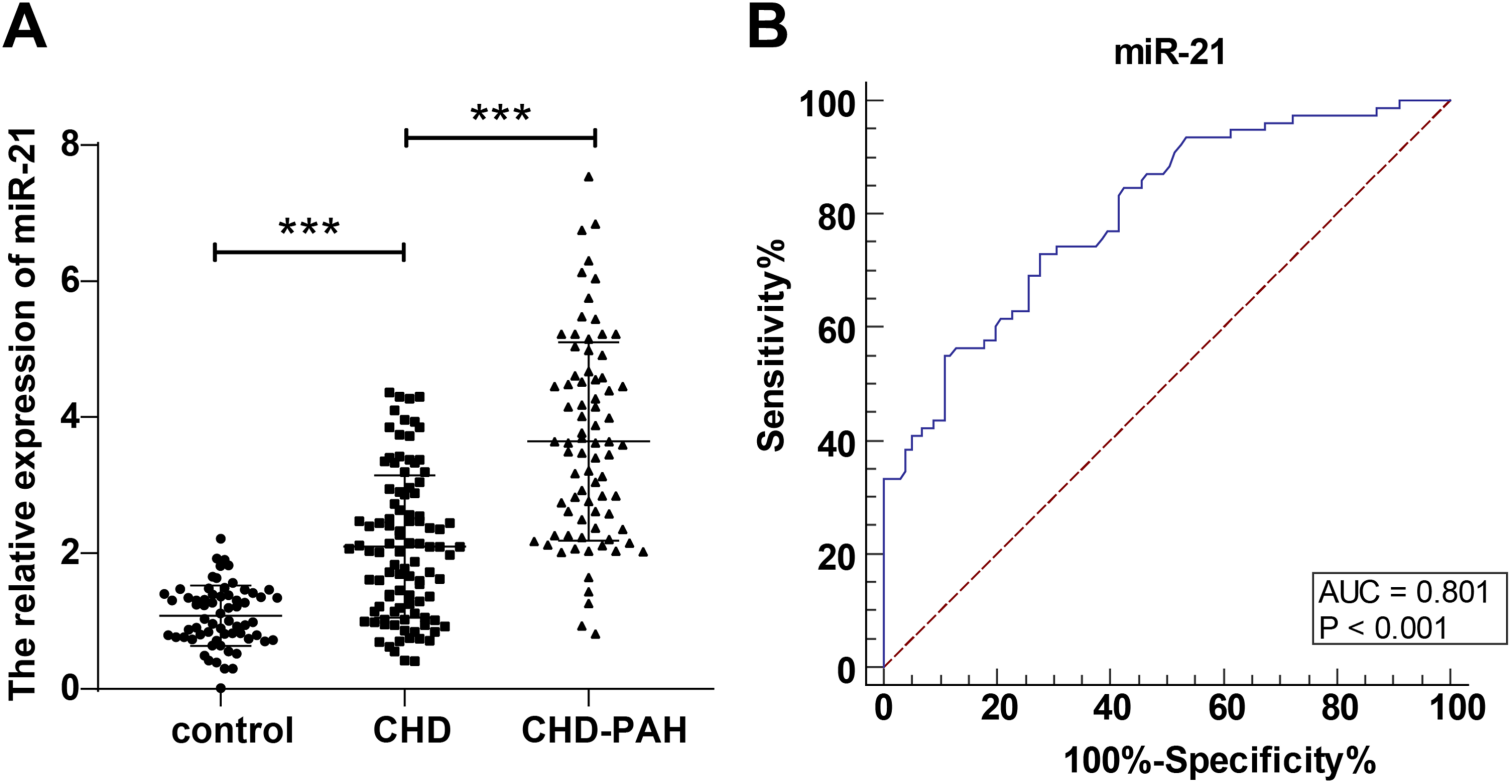
Change in serum *miR-21* level in the three groups and predictive value of serum *miR-21* for the occurrence of PAH in children with CHD. **(A)**: comparison of relative expression of serum *miR-21* in the three groups; **(B)**: predictive value of *miR-21* for the occurrence of PAH in children with CHD based on plotted ROC curve. Serum *miR-21* level was detected through RT-qPCR and expressed as average ± standard deviation. One-way analysis of variance was used for comparisons among multiple groups and Tukey's multiple comparisons test for *post-hoc* test. ***P < 0.001. CHD, congenital heart disease; PAH, pulmonary artery hypertension.

Based on the comparisons of serum *miR-21* levels among control, CHD, and CHD-PAH groups, the relative expression of serum *miR-21* of the control group was (1.08 ± 0.44), and that in the children in the CHD group and CHD-PAH group was (2.10 ± 1.05) and (3.64 ± 1.46) respectively; the serum *miR-21* levels of the children in the CHD group were significantly higher than those of the control group, and those of the CHD-PAH group were higher than those of the CHD group (*P *< 0.001, Figure 1A). The predictive value of serum *miR-21* level for the occurrence of PAH in children with CHD was further evaluated. According to the plotted ROC curve, the serum *miR-21* was shown to assist in predicting the occurrence of PAH in children with CHD with an AUC of 0.801, a 95% CI of 0.735∼0.857, a cut-off value of 2.56, a sensitivity of 73.08%, and a specificity of 72.28% (Figure 1B).

### Relationship between serum *miR-21* level in children with CHD-PAH and their clinical pathological indexes

Children with CHD-PAH were categorized into *miR-21* high group and miR-21 low group according to the ROC cut-off value of *miR-21*, and the relationship between serum *miR-21* levels in children with CHD-PAH and their clinical pathological indexes were analyzed. According to the results, there was no significant difference in the serum *miR-21* levels in children with CHD-PAH who had different ages, sex, BMI, disease type, EF, HR, SBP, DBP, LVEDd, dPAP, mRAP, PVR, and CK-MB (all *P* > 0.05), but the sPAP, mPAP, and BNP levels of the children in the *miR-21* high group were significantly higher than those of the children in the *miR-21* low group, and the CI in the *miR-21* high group was lower than those in the *miR-21* low group (all *P* < 0.05, Table 3).

**Table 3:** The relationship between serum *miR-21* and clinical pathogenic indexes.

Items	*miR-21* low group (*n* = 21)	*miR-21* high group (*n* = 57)	*P*-value
Sex (Male/female)	10/11	26/31	0.875
Age (year)	6.24 ± 2.39	5.54 ± 2.28	0.243
BMI(kg/m^2^)	14.18 ± 2.01	13.17 ± 2.14	0.064
CHD type [*n*(%)]			
VSD	12 (57.14)	35 (61.40)	0.786
ASD	7 (33.33)	19 (33.33)	
PDA	2 (9.52)	3 (5.26)	
HR (beats/min)	95.81 ± 2.80	94.51 ± 3.08	0.094
SBP (mmHg)	105.71 ± 13.43	102.63 ± 11.86	0.329
DBP (mmHg)	63.95 ± 6.62	62.33 ± 6.02	0.308
EF (%)	65.81 ± 6.05	63.18 ± 7.15	0.138
LVEDd (mm)	36 (29,45)	34 (28,41)	0.409
sPAP (mmHg)	48.62 ± 10.09	57.46 ± 8.92	<0.001
mPAP (mmHg)	34.05 ± 4.74	36.78 ± 5.51	0.048
dPAP (mmHg)	26.76 ± 7.58	26.44 ± 6.44	0.852
mRAP (mmHg)	5 (3,8)	6 (3,8)	0.117
PVR (WU)	3.41 ± 1.29	3.49 ± 1.11	0.770
CI (L min^−1^m^−2^)	7.11 (4.61,8.73)	5.99 (4.43,8.72)	0.010
BNP (pg ml^−1^)	343.05 ± 129.74	424.47 ± 138.55	0.022
CK-MB (U L^−1^)	24.64 ± 4.30	25.30 ± 4.43	0.559

Measurements that conformed to normal distribution were expressed as average ± standard deviation; comparisons between two groups were conducted using an independent sample *t*-test. Non-normally distributed measurements were expressed as quartiles and Mann-Whitney *U*-test was used for comparisons between groups. Count data were expressed as case numbers and percentages, and comparisons between groups were made using the chi-square test.BMI, body mass index; CHD, congenital heart disease; VSD, ventricular septal defect; ASD, atrial septal defect; PDA, patent ductus arteriosus; HR, heart rate; SBP, systolic blood pressure; DBP, diastolic blood pressure; EF, ejection fraction; LVEDd, left ventricular end-diastolic diameter; sPAP, systolic pulmonary arterial pressure; mPAP, mean pulmonary arterial pressure; dPAP, diastolic pulmonary arterial pressure; mRAP, mean right atrial pressure; PVR, pulmonary vascular resistance; CI, cardiac index; BNP, B-type natriuretic peptide; CK-MB, creatine kinase-MB.

### Increased serum *miR-21* level was an independent risk factor for PAH in children with CHD

For accurate evaluation of the impacts of serum *miR-21* level on the occurrence and development of PAH, EF, CI, BNP CK-MB, and serum *miR-21* level were included in multi-factor logistics regression analysis. Accordingly, increased levels of BNP and *miR-21* were considered independent risk factors for the occurrence of PAH in children with CHD (Table 4).

**Table 4:** Multi-factor logistics regression analysis of the occurrence of PAH in children with CHD.

Independent variable	*P*-value	OR value	95%CI
EF	0.312	0.938	0.830∼1.061
CI	0.832	1.069	0.577∼1.980
BNP	<0.001	1.026	1.016∼1.036
CK-MB	0.122	1.164	0.960∼1.412
*miR-21*	0.034	1.928	1.051∼3.537

CHD, congenital heart disease; PAH, pulmonary artery hypertension; EF, ejection fractio; CI, cardiac index; BNP, B-type natriuretic peptide; CK-MB, creatine kinase-MB.

### Serum *miR-21* level in children with CHD-PAH assisted in predicting postoperative critical illness

**Figure 2:**
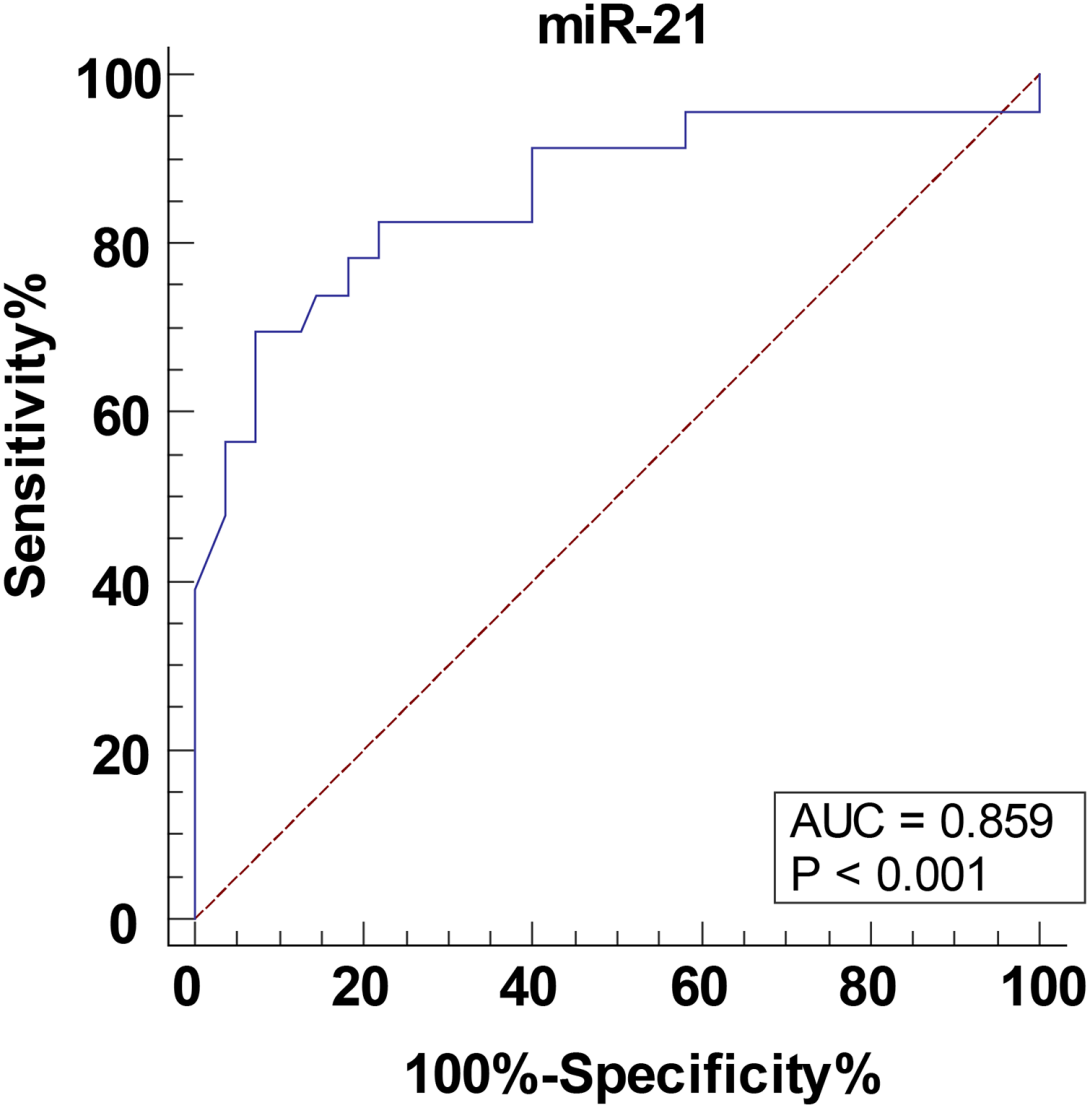
The predictive value of serum *miR-21* level for postoperative critical illness in children with CHD-PAH. The predictive value of serum *miR-21* for the occurrence of postoperative critical illness in children with CHD-PAH analyzed through the ROC curve. CHD, congenital heart disease; PAH, pulmonary artery hypertension.

The children were further divided into the non-critical group (>80 points), critical and very critical group (≤80 points) and the ROC curve was plotted to analyze the predictive value of serum *miR-21* level for postoperative critical illness in children with CHD-PAH. As shown in Figure 2, the ACU of serum *miR-21* assisting in predicting postoperative critical illness in children with CHD-PAH was 0.859, the 95% CI was 0.762∼0.927, the cutoff value was 4.55, the sensitivity was 69.57% and the specificity was 92.73%.

### Increased serum *miR-21* level was an independent risk factor for postoperative critical illness in children with CHD-PAH

Sex, age, BMI, HR, SBP, DBP, sPAP, mPAP, dPAP, EF, CI, PVR, BNP, CK-MB, and serum *miR-21* level were included in single-factor logistics regression analysis, and the age, BMI, sPAP, mPAP, EF, CI, PVR, BNP, CK-MB and *miR-21* level of children with CHD-PAH were considered as independent risk factors for postoperative critical illness. Then, indexes with *P* < 0.05 in the single-factor logistics regression analysis were used as independent variables for multi-factor logistics regression analysis, which demonstrated that higher serum *miR-21* level was an independent risk factor for postoperative critical illness in children with CHD-PAH (Table 5).

**Table 5:** Multi-factor logistics regression analysis for the occurrence of postoperative critical illness in children with CHD-PAH.

Independent variable	*P*-value	OR value	95%CI
sPAP	0.047	1.366	1.004∼1.858
mPAP	0.221	0.788	0.538∼1.154
EF	0.097	0.624	0.357∼1.090
CI	0.168	0.288	0.049∼1.690
PVR	0.100	4.746	0.742∼30.354
BNP	0.630	1.003	0.992∼1.014
CK-MB	0.199	1.271	0.881∼1.833
*miR-21*	0.041	6.371	1.080∼37.580
Age	0.126	0.364	0.099∼1.331
BMI	0.889	1.049	0.537∼2.047

CHD, congenital heart disease; PAH, pulmonary artery hypertension; EF, ejection fraction; sPAP, systolic pulmonary arterial pressure; mPAP, mean pulmonary arterial pressure; PVR, pulmonary vascular resistance; CI, cardiac index; BNP, B-type natriuretic peptide; CK-MB, creatine kinase-MB.

## Discussion

CHD-PAH occurs predominantly in patients with uncorrected CHD (22). Our study found that children with CHD-PAH had elevated sPAP, mPAP, dPAP and PVR, which are common indexes used for the diagnosis of CHD (23), indicating that the occurrence of PAH may deteriorate the condition of children with CHD. This study mainly explored the variation of serum *miR-21* levels and its significance in children with CHD-PAH, and demonstrated an increase in serum *miR-21* levels in children with CHD-PAH. The findings of this study can assist in predicting the occurrence of PAH in children with CHD and the severity of conditions post-operation.

*miR-21* has been identified as an upregulated transpulmonary exosomal miRNA in patients with CHD-PAH (17). In patients with hypertensive heart disease, *miR-21* was also detected to be upregulated, and suppressing *miR-21* attenuated hypertrophic stimulation-triggered cardiac remodeling in angiotensin II-treated neonatal rats (24). Our study first observed *miR-21* upregulation in children with CHD-PAH compared to children with CHD. *miR-21* was also identified to assist in the occurrence of PAH in children with CHD, with a sensitivity of 73.8% and a specificity of 72.28%. PAH is a fatal disease with multiple pathophysiological characteristics such as pulmonary vasoconstriction, inflammation, right ventricular hypertrophy and right ventricular systolic pressure (11). In *miR-21* knockout mice, Nano-li-stimulated pulmonary inflammation, damage, and fibrosis appeared to be mitigated (25). This study also found higher sPAP, mPAP and BNP, as well as lower CI in patients with higher *miR-21* expression. The results of the multi-factor logistics regression analysis is a further reminder of how important *miR-21* is for the prediction of the occurrence of PAH. Through the analysis, *miR-21* was identified as an independent risk factor for the occurrence of PAH in children with CHD, along with BNP, which is a biomarker secreted by cardiomyocytes in response to an increase in ventricular wall stress (26). In heritable PAH and idiopathic PAH, the critical breakthrough over the past decades is that bone morphogenetic protein (BMP) signaling reduction is caused by pathogenic mutations of BMP receptor 2 (BMPR2), and triggers proliferation and anti-apoptosis of pulmonary artery smooth muscle cells; however, CHD-PAH showed the lowest level of BMPR2 mutation but commonly suppressed BMP cascade in lungs (27). BMP-7 overexpression in diabetic mice significantly reduced *miR-21* and elevated Smad7, restrained Smad3 activation, and mitigated epithelial-mesenchymal transition and extracellular matrix deposition (28). BMP-4 was reported to activate *miR-21*, which inhibited its downstream target Dedicator of cytokines protein family to result in pulmonary vascular smooth muscle cell contractility (29, 30). The above studies imply the implication of *miR-21* in vascular contractility and fibrosis, but also hint at gene regulation functions of *miR-21*. *miR-21* may be activated by BMP and then regulate its downstream targets to affect PAH; however, due to time and budget constraints, the specific molecular mechanisms of *miR-21* in CHD-PAH remain to be investigated in the future.

In case of CHD-PAH, medication can stabilize the condition but fail to cure the PAH, and correction of the anatomical deformity is recommended as soon as possible; however, children still face the risks of severe hypoxemia, reactive PAH, etc. in the early postoperative period (31). Postoperative PCIS is a tool frequently used for clinical evaluation of postoperative crisis in children (32), by which the condition can be evaluated through the frequency of score increase and interventions can be deployed dynamically. However, the score system is an early estimation formula under no-condition state. Therefore, there is a growing realization that non-invasive and effective biomarkers must be determined for clinical practice, and it is imperative for early individual evaluation of postoperative crisis in children with PAH. This study demonstrated that serum *miR-21* level can assist in predicting postoperative critical illness in children with CHD-PAH. Further multi-factor logistics regression analysis determined upregulated serum *miR-21* expression as independent risk factor of the occurrence of postoperative critical illness in children with CHD-PAH.

This study is a retrospective single-center analysis with a small sample size. The results obtained should be verified with a larger sample size in the future. Moreover, miR-21 serves effectively as a biomarker for diagnosing pulmonary hypertension (PH), enabling the differentiation between World Health Organization group 1 PH, group 2 PH, and group 3 PH (33). The relationship between miR-21 and CHD-PAH has been extensively investigated in various studies (17, 34). Previous research in animal models has revealed a biphasic regulation on cardiac remodeling and cardiomyocyte apoptosis under pulmonary hypertension triggered by the up-regulation of miR-21 at the early phase (right ventricular hypertrophy) and its down-regulation at the late phase (right ventricular dysfunction) (18). Notably, in a sheep model of pressure overload-induced pulmonary hypertension, miR-21 was identified as a crucial contributor to right ventricular hypertrophy and dysfunction (19). A prior study has demonstrated that miR-21 influences pulmonary hypertension via the TGF-*β*1/Smad2 pathway (35). Additionally, miR-21 has been implicated in the occurrence and development of hypoxic pulmonary hypertension by modulating DDAH1 expression (36). Despite these findings, the specific biological mechanism by which miR-21 affects CHD-PAH is still of high research value. Additionally, the specific pathogenesis of miR-21 affecting CHD-PAH in the current study remains incompletely understood. Therefore, additional experiments are warranted to elucidate the potential pathogenesis in subsequent investigations. Our team will further explore the molecular mechanisms of *miR-21* upregulation in CHD-PAH by developing a multi-center prospective study and enlarging the sizes of samples and matched controls to increase the sensitivity of tests and the credibility of results.

## Data Availability

The original contributions presented in the study are included in the article/Supplementary Material, further inquiries can be directed to the corresponding author.
